# Chicago Medical Society

**Published:** 1867-01

**Authors:** 


### PROCEEDINGS OF MEDICAL SOCIETIES.

### CHICAGO MEDICAL SOCIETY,

### PATHOLOGICAL SPECIMENS OF THE HEART.

Dr. Ross presented to the Society, for examination, five hearts which had been removed from subjects at the County Hospital. The history of each case with the diagnosis, as made previous to the *post mortem* examination, treatment, and abnormal appearances, made a most interesting and instructive contribution.

Case I. M. C., male, *æt.* 45 years, had suffered for twenty years from repeated attacks of rheumatism, apparently induced by exposure. He had, however, been able to follow his occupation till within six months previous to entering the hospital, although he had for several years suffered much from shortness of breath on exertion.

On admission to the hospital, there was labored breathing; inability to recline; œdema of lower limbs, and very great exhaustion. Dullness on percussion over the cardiac region, disclosed extensive hypertrophy of the heart; a loud blowing murmur with the first sound, heard most distinctly over the apex, and propagated downwards and to the left, disclosed insufficiency of the mitral valves; a murmur with the second sound, loudest at the base, and propagated upwards, showed thickening of the aortic valves. Death occurred two weeks after admission to the hospital.

The heart was found to be hypertrophied, weighing 16 ounces. The pericardium contained 4 ounces of fluid, and was adherent, in places, to the heart. The mitral valves were so thickened by the presence of calcareous deposits, that they resembled a large tumor with a narrow, slit-like opening through it. The aortic valves were also much thickened. The gall-bladder contained a large number of concretions.

Case II. E. L., *æt.* 65. Had suffered many years from rheumatism. The general symptoms, on entering the hospital, were similar to those in the case just described. There was a very loud friction sound, with regurgitation, heard with the first sound of the heart.

He was so reduced in strength that he survived but nine days. The heart was found hypertrophied, weighing 25½ ounces. The mitral valves were much thickened, reducing the opening to the form of a mere slit. The other valves were normal. The pericardium contained 10 ounces of fluid, with extensive fibrinous exudation. The spleen weighed but 2 ounces.

Case III. J. O. L., male, *æt.* 19 years. Had long suffered from severe attacks of intermittent fever. He had become much reduced and anæmic, with loss of appetite; limbs œdematous; there were symptoms, on percussion, of hypertrophy of the heart. There was severe pain in the frontal region of the head. The patient died comatose on the fifth day after entering the hospital.

At the autopsy, there was found upon the upper and lateral surfaces of the brain, extensive fibrinous deposits; the convolutions of the brain were adherent. The heart was hypertrophied, without valvular disease, and weighed 13 ounces. The spleen weighed 37 ounces.

CASE IV. P. T., *æt.* 35 years, male. Entered the hospital in an advanced stage of phthisis pulmonalis, with hypertrophy or dilatation of the heart, and insufficiency of the aortic valves. The autopsy revealed very extensive and remarkable dilatation of the right auricle, and slight dilatation of the ascending aorta. The tricuspid valves were thickened and hard; the semi-lunar valves of the aorta thickened and closed imperfectly. mitral valves were normal.

CASE V. M. M., *æt.* 35 years, male. Had a severe attack of rheumatism ten years since. Two years ago, he first began to experience difficulty in breathing. On entering the hospital he was suffering from great dyspnœa, cough, and anasarca of lower limbs. He survived but seventeen days. The heart was hypertrophied, weighing 17 ounces; the mitral valves were thickened, their edges being united to such an extent as to reduce the opening between them to a narrow slit; the other valves were normal.

### SULPHURIC ACID IN VARIOLA AND ERYSIPELAS.

Dr. O. Smith gave the history of a case of small-pox in a child 3 years of age, in which the eruption in its earliest stages completely covered the body, as in the confluent form of the disease. There was violent fever, with other symptoms peculiar to the disease.

Sulphuric acid and water, sweetened so as to resemble lemonade, was given freely to the child. In consequence of the intense thirst, very large quantities of the solution were taken. In twelve hours, the fever entirely subsided. But twelve large pustules were formed on the body, the rest of the eruption disappearing before coming to maturity. The subsequent symptoms were very mild.

Dr. Smith regards sulphuric acid, administered freely as just described, almost a specific in erysipelas, and far superior to the muriated tincture of iron.

### USE OF A CONCAVE REFLECTOR IN EXAMINATIONS OF THE EAR.

Dr. Holmes called the attention of the Society to the use of this instrument in examinations of the external meatus, as recommended in the excellent work of Von Trœltsch on diseases of the ear, translated by Dr. Roosa, Aural Surgeon to the New York Eye Infirmary. The instrument is a concave reflector, three inches in diameter, with a focal distance of four inches, and is contained in a metallic frame, with a suitable handle. In the centre of the reflector, a small disc of the amalgam is removed, as in the small concave reflector of the ophthalmoscope, to allow the rays of light from the illuminated ear to enter the observer's eye.

By means of this instrument, the deeper portions of the external meatus can be distinctly seen, even in a cloudy day, or by the light of a candle in a darkened room. The patient can be examined while lying in bed, if necessary, as well as in an erect position.

### CASE OF CEREBRO-SPINAL MENINGITIS.

Dr. Hatch read the history of a fatal case of this disease, in which the patient, male, 21 years of age, had suffered, for two weeks previous to seeking medical aid, from headache, languor, loss of appetite, and feverishness, although he had followed his daily occupation.

At the first visit of Dr. H., the patient had apparently just passed through the different stages of a violent congestive chill, which left him with a high fever; with very frequent pulse and respiration; nausea and vomiting; great restlessness; exalted sensibility of the surface; and a terrible headache, with dullness of perception. These symptoms were accompanied, in a few hours, by coma and general muscular stiffness, which was especially marked about the neck. Death occurred in about 48 hours, soon after which the peculiar spots of the disease appeared on the surface.

### ELGIN MILK CONDENSING COMPANY.

Dr. Hamill reported the observations made by himself and Dr. H. A. Johnson, during a visit to the "Elgin Milk Condensing Company," which were of a very satisfactory character. The whole process, from the delivery of the milk by the dairymen, until it was prepared in its condensed form to be delivered to consumers, was exhibited to them. The integrity of the parties, the scrupulous care with which the milk is received, the cleanliness and sweetness of every vessel through which it passes, are guarantees to the public for its good qualities. It is recommended to the profession and the community for the following reasons:—

1st. It is procured from healthy cows, that are not fed with slops from the distilleries, and are not kept, during any part of the year, in dark, close, and dirty barns.

2d. The process in no way deteriorates the quality of the milk, and its bulk is diminished, making its transportation easy.

3d. It is of uniform strength, requiring three parts of pure water to one part of condensed milk to bring it to the standard of good fresh milk. There is no drug or chemical used or mixed with it.

And, lastly, it can be kept for a much longer time, at any season of the year, from becoming sour, if proper care is taken.

It is the pecuniary interest of the company to use good milk for condensing, and none other; this furnishes an additional guard over the purity of the article.

In consideration of these facts, it is especially adapted to the nourishment of infants who are deprived of a mother's care, or of good milk; if it supplies no other want, it will confer an unlimited blessing on this dependent class of human beings.

The following letter from Prof. Johnson to Dr. H., will be read with interest by all, and needs no comment:—

DR. HAMILL—*Dear Sir:*—I received from Mr. Hinckley a specimen of the milk, condensed at Elgin in our presence, and have subjected it to an examination, for the purpose of ascertaining what changes have taken place in its constitution. I

added to one part of the "condensed milk" three parts of water, mixing well. The sp. grav. was then 1030, water being 1000. The casein did not differ, so far as I could judge, from that of good uncondensed milk. The amount of cream was *within* the range of good new milk. The corpuscles, as seen under the microscope, to a *very limited* extent, were broken, and in size, were somewhat more variable than those of good uncondensed milk, the effect, I presume, of the mechanical agitation during the process of condensation. The taste of the milk, after condensation and dilution, as compared with specimens of the same milk before condensation, was somewhat richer, giving the impression of more cream, due, I think, to the rupture, as previously stated, of the envelopes of some of the corpuscles. This, while it gives a richer taste, in no way impairs the quality of the milk. I used in my examination, as a standard of comparison, the new milk from my own cow, a healthy animal, yielding, as we think, milk of an excellent quality. This process in no way injures the quality of the milk, while it diminishes the bulk for transportation; is clean; is not likely to be adulterated, and may be kept without change for a much greater length of time. I take pleasure in saying that, in my judgment, the public may rely upon it implicitly, and I trust that the gentlemen interested may find sufficient encouragement to induce them to continue its preparation.

In conclusion, I beg leave to suggest, that, as in all milk adulterated, whether with water alone, or with water and an admixture of other ingredients, the cream is invariably diminished in relation to its bulk or quality, that the galactometer may be advantageously resorted to as a means of examination in cases of suspected falsification. This instrument consists of two glass tubes, of considerable size, connected by a smaller tube. This latter has a graduated scale attached. The instrument is filled with milk up to the small tube, a portion of acetic acid is added; the two well shaken together for a minute or two, when the butter, liberated by the acid, rises to the surface and fills the smaller tube. If gently warmed it will become limpid, and the amount will be indicated by the graduation of the smaller tube. If it be desirable to determine the proportion of cream to the whole amount of milk, the capacity of the instrument and the value of the divisions on the scale should be determined. If, for instance, the capacity of the larger tube be five times that of the smaller, and the tube be divided into ten equal parts, it would be easy to determine, within a very small fraction, the relative proportions of cream in the milk with which the instrument is charged.

This process does not give, it is true, a scientific analysis of milk, but indicating, as it does, the amount of cream, its results are sufficient for all practical purposes.

Very truly, yours,

H. A. JOHNSON.

611 Wabash Avenue, December 28th, 1866.

Prof. Davis, after stating that he was decidedly opposed to the custom of recommending, by resolution in medical societies, wines and other preparations, either medicinal or alimentary, from the fact that the articles sold to the public were too often very inferior in quality to the specimens presented to the societies for examination, added that in this case, however, from the importance of the preparation, as an article of food especially for infants, and from the fact that there were many reasons why it would be for the interest of the manufacturers to furnish pure milk, he would offer the following resolution:

*Resolved*, That the great importance of having milk pure, uniform in quality, and capable of remaining sweet longer than that ordinarily distributed directly from the dairy, makes the condensed milk furnished by the Elgin Milk Condensing Company an article of great value to the community, and one which we freely recommend for general use, and especially for use in feeding children.

Unanimously adopted. Several members, who had observed the very favorable results from its use, not only in their own families, but also in the cases of their patients, spoke warmly in recommendation of this milk.

